# Circulating microRNAs: next-generation biomarkers for early lung cancer detection

**DOI:** 10.3332/ecancer.2012.246

**Published:** 2012-03-06

**Authors:** F Bianchi, F Nicassio, G Veronesi, PP di Fiore

**Affiliations:** 1 Department of Experimental Oncology, European Institute of Oncology, Milan, Italy; 2 Division of Thoracic Surgery, European Institute of Oncology, Milan, Italy; 3 IFOM, The FIRC Institute for Molecular Oncology Foundation, Italy; 4 Dipartimento di Medicina, Chirurgia ed Odontoiatria, Universita’ degli Studi di Milano, Milan, Italy

## Abstract

Early diagnosis of lung cancer by low-dose computed tomography is an effective strategy to reduce cancer mortality in high-risk individuals. However, recruitment of at-risk individuals with asymptomatic lung cancer still remains challenging. We developed a minimal invasive serum test, based on the detection of circulating microRNAs, which can identify at-risk individuals with asymptomatic early stage non-small cell lung carcinomas with 80% accuracy.

## Background and discussions

Lung cancer is the leading cause of cancer-related deaths in the developed world, accounting for 156,940 estimated deaths in 2011 in the US alone [1]. The lethality of lung cancer is primarily due to the lack of effective strategies for early detection, at a stage when the tumour would still be curable by surgery. However, detection of early stage lung cancer is challenging due to its frequent absence of symptoms [2]. This explains the urgent need for early detection screening programs for lung cancer in high-risk individuals, i.e. current or former heavy smokers (>20 packs/year) aged 50 years or above.

Low-dose spiral computed tomography (LD-CT) is an effective detection method for small lung nodules (even less than 5 mm) that subjects patients to low radiation exposure with no contrast medium, has limited costs and requires only a few seconds of execution [3,4]. The recent report from the large randomised *National Lung Screening Trial* has demonstrated a mortality reduction of 20% in the arm screened by LD-CT compared with the arm screened by chest x-ray [5]. However, recruitment of at-risk individuals with no symptoms of disease is still demanding.

In such a scenario, the development of blood tests able to detect the presence of lung cancer might form the cornerstone for successful population-based cancer screenings. In recent years there have been many attempts to identify serum/plasma biomarkers for lung cancer detection. Some studies have been based on detection through an enzyme-linked immunosorbent assay (ELISA) of circulating tumour-associated antigens (TAA) such as p53, NY-ESO-1, CAGE, GBU 4-5, annexin, or SOX2, which displayed an overall good specificity and sensitivity (40% sensitivity, 90% specificity [6]). Others have relied on the detection of circulating cancer cells in the blood of patients with metastatic tumours [7]. Such chip-based tests show sensitivity and specificity in detecting cancer cells that is close to 100% [8], warranting further investigations into the applicability of the detection circulating cancer cells at earlier stages of disease.

Recently, we and others have identified a subset of circulating microRNAs (miRNAs) accurate enough to detect symptomatic lung cancer [9–13] and, more importantly, LD-CT-detected asymptomatic lung cancer [14,15]. The detection of circulating miRNAs may be a valid alternative to LD-CT for the early diagnosis of cancer [16], since these tiny molecules are only marginally affected by degradation [17] and can be easily quantified by real-time PCR, a method routinely used in the clinic. In a series of experiments described in more detail elsewhere [14], we used real-time PCR to identify an expression profile of miRNAs extracted from the sera of participants enrolled in a large single-centre observational study (COSMOS study [18]). Among the 147 miRNAs detected by real-time PCR, 34 showed differences in expression in asymptomatic LD-CT-detected lung adenocarcinoma versus normal sera. We developed a multivariate risk-predictor algorithm based on the weighted linear combination of the 34-miRNA expression levels. When the predictor was tested on an independent cohort of patients with asymptomatic LD-CT-detected lung cancer, it displayed an overall accuracy of 80% (sensitivity 71%, specificity 90%; AUC 0.89). Furthermore, through a series of additional experiments, we also showed that the risk-predictor was able to distinguish between LD-CT-detected benign nodules and frankly malignant disease. This very important finding demonstrates the specificity of the test and highlights its utility in the clinic, because of the relative high number of benign lung nodules detected by LD-CT screening [19,20].

**Figure 1:**
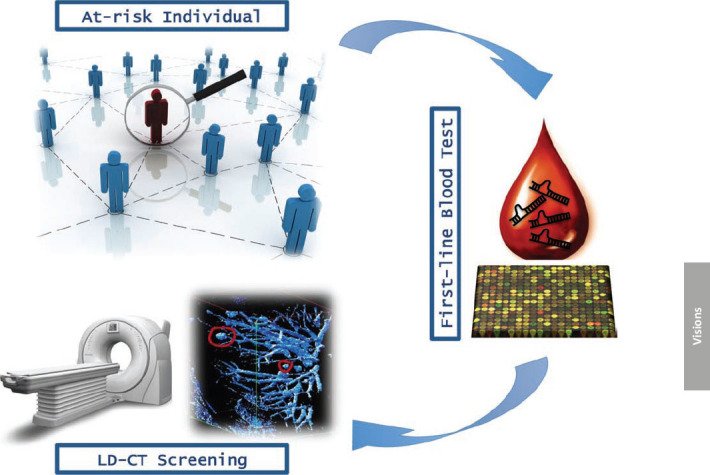
Overview of lung cancer early detection mass population screening.

The simplicity of the procedure (it is minimally invasive, requiring <1 ml of serum) and its relatively low cost (based on standard real-time PCR) should encourage population compliance to large-scale screening programs, thus accelerating its application in the clinic as a ‘first line screening test’ to identify those high-risk individuals who should undergo further testing, including by LD-CT (Figure 1). However, unresolved questions still remain (as for all newly proposed genomic blood tests) regarding tumour specificity.

We tried to initially address this issue by including in our analysis also a group of patients with invasive ductal breast carcinoma or with benign breast fibroadenoma, as a control. The test resulted to be negative in this additional group of patients, thus demonstrating a certain specificity of the 34-miRNA test for lung cancer detection [14].

Lastly, a critical issue relates to the origin of serum circulating miRNAs and their biological function. The current model implies that miRNA are released in membrane-bound vesicles [21–23], which protect them from blood RNAse activity [24–26]. Such kind of vesicles could be either exosomes, 50–90 nm vesicles of endocytic origin arising by inward budding of multivesicular bodies and released by exocytosis [27], or microvesicles, larger (up to 1 um) membrane-bound particles generally derived by membrane shedding of several cell types [24,28].

Strikingly, tumour cells appear to communicate through exosomes with immune cells, leading to immune suppression [29,30]. It is tempting to speculate that lung cancer cells might reprogram the tumour microenvironment, perhaps by altering the expression patterns of surrounding cells, including those of the immune system, through miRNAs contained in exosomes. Undoubtedly, future studies will shed more light on the biological functions of circulating miRNAs and their role, if any, in cancer progression. Such studies will allow researchers to design completely novel strategies for lung cancer therapy.
